# Long non-coding RNA DHRS4 antisense RNA 1 inhibits ectopic endometrial cell proliferation, migration, and invasion in endometriosis by regulating microRNA-139-5p expression

**DOI:** 10.1080/21655979.2022.2060781

**Published:** 2022-04-12

**Authors:** Xuan Cui, Shisan Zhou, Yongtao Lin

**Affiliations:** a School of Nursing and Midwifery, Jiangsu College of Nursing, Huai’an, China; b Department of Anesthesiology, Huaian Maternity and Child Health Care Hospital, Huai’an, China; c Department of Nephrology, Affiliated Huai’an Hospital of Xuzhou Medical University, Huai’an, P.R. China

**Keywords:** DHRS4 antisense RNA 1, microRNA-139-5p, arrestin domain containing 3, endometriosis, endometrium stroma cells

## Abstract

Endometriosis is an estrogen-dependent chronic gynecological syndrome. Recent studies have shown that long non-coding RNAs participate in the pathogenesis and development of endometriosis. This study aimed to explore the mechanisms of DHRS4 antisense RNA 1 (DHRS4-AS1) in endometriosis. Dual-luciferase reporter assays were conducted to determine the relationship between DHRS4-AS1, microRNA (miR)-139-5p, and arrestin domain-containing 3 (ARRDC3). Furthermore, the expression of DHRS4-AS1 and miR-139-5p in ectopic endometrial stromal cells (EC-ESCs) and endometriosis tissues was examined using reverse transcription-quantitative polymerase chain reaction (RT-qPCR). Additionally, 3-(4,5-dimethyl-2-thiazolyl)-2,5-diphenyl-2-H-tetrazolium bromide (MTT), flow cytometry, and Transwell assays were performed to evaluate the proliferation, apoptosis, and migration and invasion of EC-ESCs, respectively. Western blotting and RT-qPCR were further utilized to determine cleaved-Caspase 3, Caspase 3, and matrix metalloproteinase 9 (MMP-9) expression levels. Compared with the EN group, DHRS4-AS1 levels were lower and miR-139-5p levels were higher in EC-ESCs and tissues obtained from patients with endometriosis. Functional assays validated that DHRS4-AS1 targets miR-139-5p, with ARRDC3 being a downstream target of miR-139-5p. Rescue experiments demonstrated that DHRS4-AS1 inhibited EC-ESC proliferation, migration, and invasion, but promoted apoptosis, by targeting miR-139-5p in endometriosis. cleaved-Caspase3 expression level and the cleaved-Caspase 3/Caspase 3 ratio increased, while the expression levels of MMP-9 decreased, after transfection with DHRS4-AS1 overexpression plasmids; however, the effects induced by DHRS4-AS1 overexpression could be partially reversed by co-transfection with the miR-139-5p mimic. The current study demonstrates that the DHRS4-AS1/miR-139-5p/ARRDC3 axis participates in the regulation of EC-ESC function.

## Graphical abstract

**Figure:**
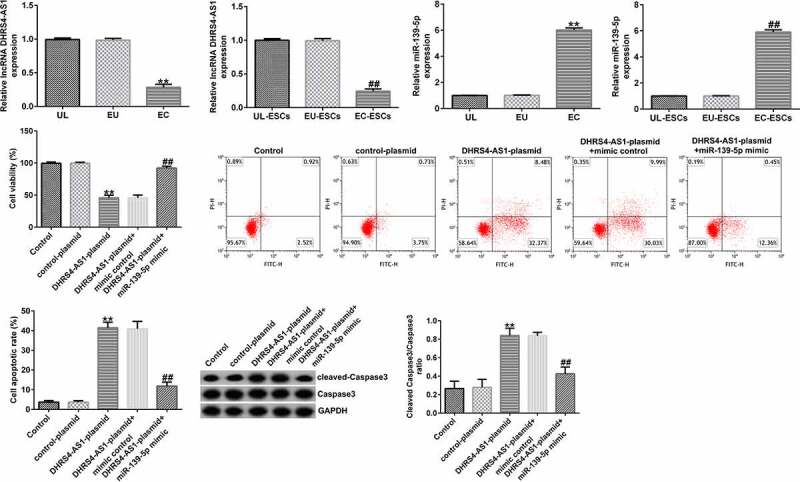

## Introduction

Endometriosis constitutes the growth of active endometrium outside the uterine cavity [1]. It is an estrogen-dependent chronic gynecological disorder with cellular dysfunction [2]. The ubiquity of endometriosis has been estimated to be ~10–15% in women of reproductive age, and it increases to 70% in patients with chronic pelvic pain [3]. Periodic bleeding of the ectopic endometrium (EC) leads to inflammatory infiltration within the affected area, producing adhesions and scarring and causing a series of symptoms, such as dysmenorrhea, chronic pelvic pain, and infertility [4]. Similar to malignant tumors, endometriosis can grow widely and invasively [5]. Currently, laparoscopy is regarded as the gold standard for the diagnosis of endometriosis [6]. However, the invasiveness of laparoscopy, coupled with a lack of sensitive and specific biomarkers, has resulted in an average time of 6–7 years from symptom onset to diagnosis [7]. A delayed diagnosis may have an unfavorable impact on disease progression. Clinical treatments for endometriosis include pharmacological and surgical interventions, with surgical and adjunctive pharmacological treatments being the most common [8]. However, the effect of treatment is often unsatisfactory, with patients exhibiting high recurrence and low cure rates [9]. Therefore, the further in-depth study of the pathogenesis and pathology of endometriosis is necessary to explore and develop effective clinical treatment methods and evaluate treatment effects and prognosis.

Although endometriosis is categorized as a benign disease, it also displays ordinary features of malignant tumors, such as unlimited growth, infiltration and destruction of surrounding tissues, and local or distant metastasis [10]. In addition, the migration and invasion characteristics of ectopic endometrial stromal cells (EC-ESCs) are almost identical to those of malignant cancer cells [11,12]. To date, the molecular mechanisms underlying the proliferation, migration, and invasion characteristics of EC-ESCs remain unclear.

Recently, long non-coding RNAs (lncRNAs) have been shown to perform vital functions in various diseases, including endometriosis [13,14]. Various lncRNAs have been reported to be involved in the pathogenesis and progression of endometriosis. For example, Mai et al. [15] revealed that long intergenic non-protein coding RNA 1541 decreased in ectopic tissues with endometriosis, inhibiting ESC proliferation, migration, and invasion, in addition to the microRNA (miRNA/miR)-506-5p/Wnt/β-catenin pathway. Feng et al. [16] also observed decreased expression of lncRNA MALAT1 in human ESCs, promoting ESC apoptosis via the miR-126-5p/CAMP responsive element binding protein 1/PI3K/AKT signaling axis. However, to the best of our knowledge, the role of the lncRNA DHRS4 antisense RNA 1 (DHRS4-AS1) in EC-ESCs and endometriosis remains unknown.

Furthermore, miRNAs are non-coding, single-stranded RNAs consisting of only 20–23 nucleotides [17]; they are commonly found in eukaryotic organisms that play essential roles in various diseases [18]. By binding to the 3’-untranslated regions of mRNAs, miRNAs directly induce the degradation of target mRNAs, thus participating in the regulation of a variety of cellular functions [19]. It has been determined that miRNAs are ideal biomarkers that exhibit high stability, specificity, and sensitivity and can be utilized for the early diagnosis and prognosis of various diseases [20,21]. In a previous study, Rekker et al. [22] observed that miR-139-5p levels were significantly augmented in EC-ESCs compared to eutopic cells. However, the underlying mechanisms of miR-139-5p in regulating endometriosis development remain to be clarified.

Additionally, lncRNA DHRS4-AS1 has been reported to perform essential functions in regulating cancer cell proliferation, migration, and invasion [23–25]. The lncRNA DHRS4-AS1 may play a vital role in regulating the proliferation, migration, and invasion characteristics of EC-ESCs, thus participating in endometriosis. Through bioinformatic analysis, we identified the binding sites between lncRNA DHRS4-AS1 and miR-139-5p. Therefore, we hypothesized that DHRS4-AS1 may be involved in the occurrence and development of endometriosis by regulating miR-139-5p. And this study investigated the abnormal expression and underlying mechanism of DHRS4-AS1 and miR-139-5p in endometriosis to understand the diagnosis and treatment of endometriosis.

## Materials and methods

### Specimen collection

This study was approved by the Ethics Committee of the Affiliated Huai’an Hospital of Xuzhou Medical University and performed in compliance with the Declaration of Helsinki [26]. Prior to enrollment, written informed consent was obtained from each patient. Patients with endometriosis (n = 15) or uterine leiomyoma (UL; n = 15) who underwent surgical treatment at the Affiliated Huai’an Hospital of Xuzhou Medical University between April 2017 and December 2020 were enrolled in the current study. Endometriosis was diagnosed by histopathological examination. Suspicion for endometriosis was raised by symptoms and ultrasound examination by transvaginal ultrasound and confirmed by histopathological examination. The golden standard for endometriosis detection is histological confirmation. Ectopic or eutopic endometrial specimens were obtained from women aged 20–45 under regular menstruation without any hormone treatment within the last six months. The inclusion criteria for endometriosis patients were as follows: i) patients in the mid-to-late proliferative phase of the disease [27], and ii) patients who had not received hormonal treatment 6 months prior to surgery. Patients with the following syndromes were excluded: i) severe liver, heart, lung, and kidney diseases; ii) pregnant or lactating women; iii) autoimmune diseases or any malignant tumors; and iv) complicated endocrine disorders or other gynecological diseases (gynecological diseases other than endometriosis).

Endometrial samples obtained from 15 patients with UL were used as the controls. The EC and eutopic endometrium (EU) were collected from 15 patients with endometriosis after surgical treatment. EC is considered an endometriotic lesion outside the cavity obtained via surgery (open or laparoscopic approach). The EU is defined as the non-pathological endometrium inside the cavity obtained through diagnostic curettage, a surgical intervention.

### Cell culture and transfection

ESCs were isolated from the EC-ESCs and eutopic endometria (EU-ESCs) of 10 patients with endometriosis, as described previously [28,29]. ESCs from endometrial samples of 10 patients with UL were also isolated. Briefly, tissue samples were digested with 0.1% type IV collagenase solution and 0.25% trypsin at 37°C for 2 h, washed three times with PBS, and dissected. ESCs were separated from epithelial cells and debris using 150- and 37.4-μm sieves. The purity of ESCs was analyzed following the third passage, and ESCs with >99% purity were selected for subsequent experiments. EC-ESCs, EU-ESCs, and EN-ESCs were cultured in F12/Dulbecco’s modified Eagle’s medium (DMEM) (Thermo Fisher Scientific, Inc.) supplemented with 10% fetal bovine serum (FBS) (Invitrogen; Thermo Fisher Scientific, Inc.) and incubated in 5% CO_2_ at 37°C.

Additionally, 293 T cells were attained from The American Type Culture Collection and cultured in DMEM (Gibco; Thermo Fisher Scientific, Inc.) supplemented with 10% FBS and 1% penicillin-streptomycin in a humidified atmosphere with 5% CO_2_ at 37°C.

The miR-139-5p mimic (5’-UCUACAGUGCACGUGUCUCCAG-3’) and mimic control (5’-UCUCCGAACGUGUCACGU-3’) were obtained from Shanghai GenePharma Co., Ltd. Full-length DHRS4-AS1 was amplified via polymerase chain reaction (PCR) and sub-cloned into the pcDNA3.1 empty vector (Guangzhou RiboBio Co., Ltd.) to construct the DHRS4-AS1 overexpression plasmid. The 100-nM miR-139-5p mimic, 100-nM mimic control, 1-µg DHRS4-AS1 plasmid, and 1-µg control plasmid were transfected into EC-ESCs using Lipofectamine® 3000 (Invitrogen; Thermo Fisher Scientific, Inc.) according to the manufacturer’s instructions. Untreated cells were used as controls.

### RNA isolation and reverse transcription-quantitative PCR (RT-qPCR) analysis

Total RNA was isolated from the tissues and cells using TRIzol® reagent (Invitrogen; Thermo Fisher Scientific, Inc.) and purified using a NanoDrop spectrophotometer. RNA was reverse-transcribed into cDNA using a cDNA synthesis kit (Takara Bio, Inc.) according to the manufacturer’s protocol. Then, qPCR analysis was conducted using a 7500 ABI Real-Time PCR system (Applied Biosystems; Thermo Fisher Scientific, Inc.) with a SYBR Green kit (Takara Bio, Inc.) under the following thermocycling conditions: 95°C for 5 min, followed by 35 cycles of 95°C for 20s, and 65°C for 45s. The experimental results obtained from the PCR were normalized to U6 or glyceraldehyde 3-phosphate dehydrogenase (GAPDH) using the 2^−∆∆Cq^ method [30]. Primer sequences were obtained from Sangon Biotech (Shanghai, China) and verified using BLAST. Primer sequences were as follows: DHRS4-AS1 forward, 5ʹ-GGAGGCTGAGGCAGGAGAAT-3ʹ, and reverse, 5ʹ-GCTAGTCTGGTCACCTCTGGAT-3ʹ; GAPDH forward, 5ʹ-CACCCACTCCTCCACCTTTG-3ʹ and reverse, 5ʹ-CCACCACCCTGTTGCTGT AG-3ʹ; miR-139-5p forward, 5ʹ-GCCTCTACAGTGCACGTGTCTC-3ʹ and reverse, 5ʹ-CGCTGTTCTCATCTGTCTCGC-3ʹ; MMP-9 forward, 5ʹ-GCATAAGGACGACGTGAATGGC-3ʹ and reverse, 5ʹ-CGGTGTGGTGGTGGTTGGAG-3ʹ; and U6 forward, 5ʹ-CTCGCTTCGGCAGCACA-3ʹ, and reverse, 5ʹ-AACGCTTCACGAATTTGCGT-3ʹ.

### Western blot assay

Protein expression was detected using western blot assay [31]. Cells were lysed in radioimmunoprecipitation assay lysis buffer, and the protein concentration was measured using a bicinchoninic acid kit (Beyotime). Subsequently, ~25 μg of protein was separated by 12% sodium dodecyl sulfate-polyacrylamide gel electrophoresis and transferred onto polyvinylidene fluoride membranes (Millipore Sigma). After blocking with 5% nonfat milk for 50 min at room temperature, the membranes were probed with the following primary antibodies at 4°C overnight (all Abcam): rabbit anti-cleaved caspase-3 (cat. no. ab2302; 1:1,000), rabbit anti-caspase-3 (cat. no. ab32351; 1:2,000), rabbit anti-MMP-9 (cat. no. ab76003; 1:1,000), and rabbit anti-GAPDH (cat. no. ab9485; 1:1,000). The membranes were then incubated with pre-adsorbed goat anti-rabbit IgG H&L (HRP) (cat. no. ab7097; 1:1,000; Abcam) for 1 h at room temperature. An enhanced chemiluminescence kit (Thermo Fisher Scientific, Inc.) was used to detect the bands, with GAPDH serving as the loading control.

### MTT assay

Cell proliferation was assessed using the MTT assay [32]. Cells were inoculated into 96-well-plates at a density of 3,000 cells/well and cultured in an incubator for 12 h at 5% CO_2_ and 37°C. After 48 h of cell transfection, the MTT reagent (10 µL/well) was added to each well and further incubated at 37°C for 2 h. Optical density was measured at 570 nm using a microplate reader. Three replicate experiments were performed for the MTT assay, after which the proliferation of each group was calculated.

### Flow cytometry analysis

Cell apoptosis was determined using the Annexin V-FITC Apoptosis Detection Kit (Beyotime), according to the manufacturer’s protocol [33]. Samples were resuspended in binding buffer and incubated with 5 μL of Annexin V-FITC and 10 μL of propidium iodide reagent at room temperature in the dark for 15 min. Flow cytometry (BD Biosciences) with Kaluza analysis software (version 2.1.1.20653; Beckman Coulter, Inc.) was used to evaluate the number of apoptotic cells.

### Transwell assay

Cell migration and invasion were evaluated using a Transwell assay [34]. After cell digestion and centrifugation at 4°C at 10,000 *× g* for 15 min, cells were resuspended in serum-free F12/DMEM medium and diluted to a density of ~ 2 × 105 cells. For migration and invasion, 200 µL of the cell suspension was carefully inoculated into the upper chamber of a Transwell insert (without or with Matrigel). A total of 700 µL of the medium containing 10% FBS was added to the lower chamber. Transwell chambers were then carefully placed into 24-well culture plates and incubated at 37°C and 5% CO_2_. After 24 h, the cell suspension within the chambers was aspirated, discarded, and washed twice with sterile PBS. Non-migratory and noninvasive cells were gently removed using cotton swabs. The remaining cells were fixed in 4% paraformaldehyde at room temperature for 30 min and then stained with 0.1% crystal violet at room temperature for 30 min, after which the stained cells were observed under a light microscope (Olympus Corporation; magnification, ×200).

### Dual-luciferase reporter assay

The potential binding sites between miR-139-5p and DHRS4-AS1 were predicted using bioinformatics software (StarBase: http://starbase.sysu.edu.cn/). Wild-type (wt) and mutant (mut) DHRS4-AS1 were constructed based on the predicted binding sites and cloned into the dual-luciferase vector pmiGLO (cat no. VT1439; YouBio Biology). Then, 293 T cells were inoculated into 96-well-plates at a density of 1 × 10^4^ cells/well and co-transfected with DHRS4-AS1-wt or DHRS4-AS1-mut and miR-139-5p mimic or mimic control using Lipofectamine® 3000 (Invitrogen; Thermo Fisher Scientific, Inc.). After co-transfection for 48 h, a dual luciferase assay kit (Promega Corporation) was used to detect the luciferase activity of the samples, in accordance with the manufacturer’s instructions. Relative luciferase activity was normalized to that of *Renilla* luciferase [35].

The potential binding sites between miR-139-5p and arrestin domain-containing 3 (ARRDC3) were predicted using bioinformatics software (TargetScan: http://www.targetscan.org/vert_72/). A dual-luciferase reporter assay was performed to verify the binding sites between miR-139-5p and ARRDC3, as described above.

### Statistical analysis

All experiments were repeated at least three times. Data are presented as mean ± standard deviation and were analyzed using GraphPad Prism software (version 6.0; GraphPad Software, Inc.). P < 0.05 was considered as the threshold for statistical significance.

## Results

**Figure 1.**
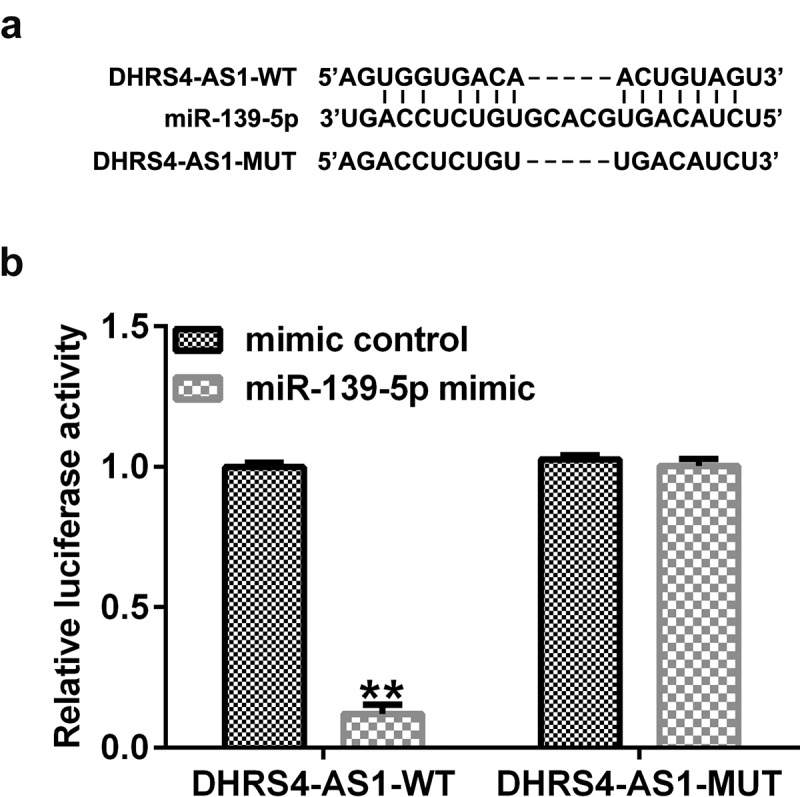
DHRS4-AS1 targets miR-139-5p. (a) The putative binding sites between DHRS4-AS1 and miR-139-5p were predicted using StarBase. (b) A Dual-luciferase reporter assay confirmed the targeted relationship between DHRS4-AS1 and miR-139-5p. **P < 0.01 vs. mimic control. DHRS4-AS1, DHRS4 antisense RNA 1; miR, microRNA.

Direct interaction between *DHRS4-AS1 sponged to miR-139-5p*. The putative binding sites of DHRS4-AS1 and miR-139-5p are shown in Figure 1a. The dual-luciferase reporter assay validated that the luciferase activity of the DHRS4-AS1-wt group was significantly reduced when co-transfected with the miR-139-5p mimic compared to co-transfection with the mimic control group (Figure 1b). However, no significant differences were observed between the DHRS4-AS1-mut groups. These results demonstrated that DHRS4-AS1 was a target of miR-139-5p.

### DHRS4-AS1 expression levels decreased, while miR-139-5p expression levels increased in endometrial tissues and cells

**Figure 2.**
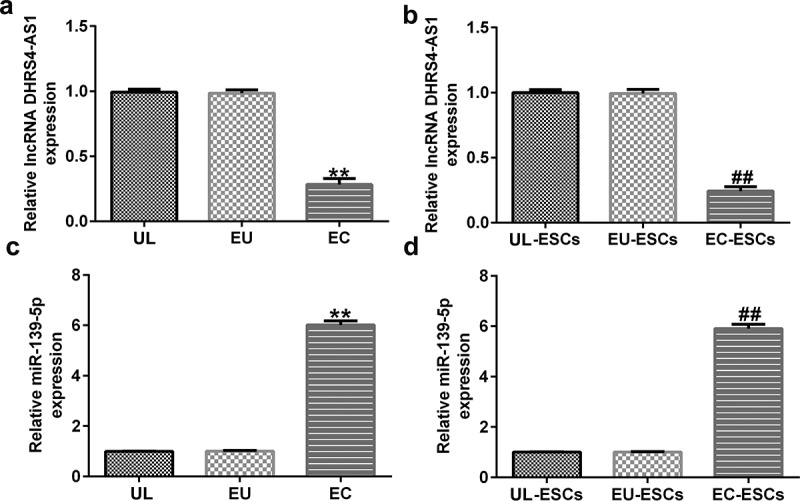
DHRS4-AS1 levels decreased, while miR-139-5p expression was upregulated, in endometriotic tissues and cells. RT-qPCR analysis was performed to quantify the expression levels of (a) DHRS4-AS1 in the EC, EU, and UL (n = 15); (b) DHRS4-AS1 in EC-ESCs, EU-ESCs, and UL-ESCs (n = 10); (c) miR-139-5p in the EC, EU, and UL (n = 15); and (d) miR-139-5p in EC-ESCs, EU-ESCs, and UL-ESCs (n = 10). **P < 0.01 vs. UL; ##P < 0.01 vs. UL-ESCs. DHRS4-AS1, DHRS4 antisense RNA 1; miR, microRNA; RT-qPCR, reverse transcription-quantitative polymerase chain reaction; EC, ectopic endometrium; EU, eutopic endometrium; UL, uterine leiomyoma endometrium as control; ESCs, endometrial stromal cells.

To confirm the roles of DHRS4-AS1 and miR-139-5p in endometriosis, aberrant expression of DHRS4-AS1 and miR-139-5p in endometrial tissues and cells was investigated. The results showed that the expression levels of DHRS4-AS1 markedly decreased (Figure 2a), while miR-139-5p expression levels increased in EC tissues relative to UL tissues (Figure 2c).

To further elucidate the fundamental mechanisms of DHRS4-AS1 and miR-139-5p in endometriosis, ESCs were isolated from EC and EU tissues. As presented in Figure 2bandd, DHRS4-AS1 expression levels significantly decreased, whereas miR-139-5p expression levels increased, in EC-ESCs compared to UL-ESCs.

### DHRS4-AS1 inhibits proliferation and promotes apoptosis of EC-ESCs by targeting miR-139-5p

**Figure 3.**
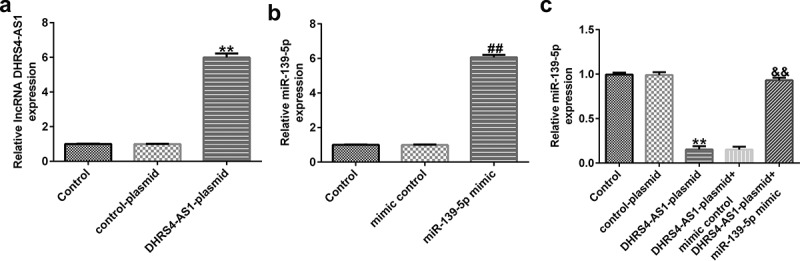
Transfection efficiency of the DHRS4-AS1 plasmid and miR-139-5p mimic in EC-ESCs. (a) Expression level of DHRS4-AS1 in EC-ESCs after transfection with DHRS4-AS1 and control plasmids was determined using RT-qPCR. (b) miR-139-5p level in the miR-139-5p-mimic or mimic control group was determined via RT-qPCR. (c) The effect of DHRS4-AS1 on miR-139-5p expression was evaluated by performing RT-qPCR. **P < 0.01 vs. control plasmid. ##P < 0.01 vs. mimic control. &&P < 0.01 vs. DHRS4-AS1 plasmid + mimic control. DHRS4-AS1, DHRS4 antisense RNA 1; miR, microRNA; EC-ESCs, ectopic endometrial stromal cells; RT-qPCR, reverse transcription-quantitative polymerase chain reaction.

To investigate the effects of DHRS4-AS1 on miR-139-5p expression in EC-ESCs, EC-ESCs were transfected with DHRS4-AS1 plasmid, miR-139-5p mimic, or DHRS4-AS1 plasmid+miR-139-5p mimic. As shown in Figure 3aandb, DHRS4-AS1 expression was profoundly augmented in the DHRS4-AS1 plasmid group compared to the control plasmid group, whereas compared to the mimic control group, miR-139-5p expression level increased after transfection with the miR-139-5p mimic. These results indicated that transfection was successful. Furthermore, DHRS4-AS1 overexpression suppressed miR-139-5p expression, whereas co-transfection with the miR-139-5p mimic antagonized DHRS4-AS1 plasmid-induced suppression of miR-139-5p expression (Figure 3c).

**Figure 4.**
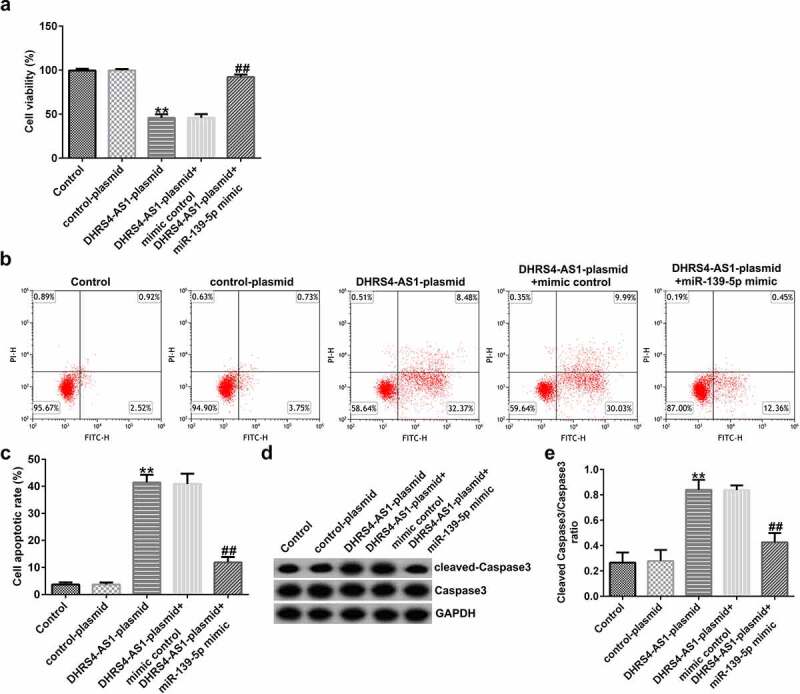
DHRS4-AS1 inhibits the proliferation and promotes the apoptosis of EC-ESCs by targeting miR-139-5p. (a) The viability of EC-ESCs was determined via MTT assay. (b and c) Apoptosis rate of EC-ESCs was evaluated via flow cytometry. (d) Expression levels of apoptosis-related proteins, including cleaved caspase-3 and caspase-3, were quantified in different groups. (e) Expression ratio of cleaved caspase-3/caspase-3 was calculated. **P < 0.01 vs. control plasmid. ##P < 0.01 vs. DHRS4-AS1 plasmid + mimic control. DHRS4-AS1, DHRS4 antisense RNA 1; EC-ESCs, ectopic endometrial stromal cells; miR, microRNA.

MTT and flow cytometry assays were conducted to investigate the effects of both DHRS4-AS1 and miR-139-5p on EC-ESC proliferation and apoptosis, respectively. As presented in Figure 4a-c, DHRS4-AS1 overexpression suppressed EC-ESC viability and enhanced apoptosis, compared to the control plasmid group; however, the effects induced by DHRS4-AS1 overexpression could be partly reversed by treatment with the miR-139-5p mimic. Similarly, after transfection with the DHRS4-AS1 plasmid, the ratio of protein expression of cleaved caspase-3 to the cleaved caspase-3/caspase-3 ratio was augmented compared to the control plasmid group (Figure 4dande). However, the effect of the DHRS4-AS1 plasmid on cleaved caspase-3 protein expression and the cleaved caspase-3/caspase-3 ratio was counteracted by co-transfection with the miR-139-5p mimic.

### DHRS4-AS1 suppresses EC-ESC migration and invasion by sponging miR-139-5p. To determine the effects of DHRS4-AS1 on cell migration and invasion, transwell assay was performed

**Figure 5.**
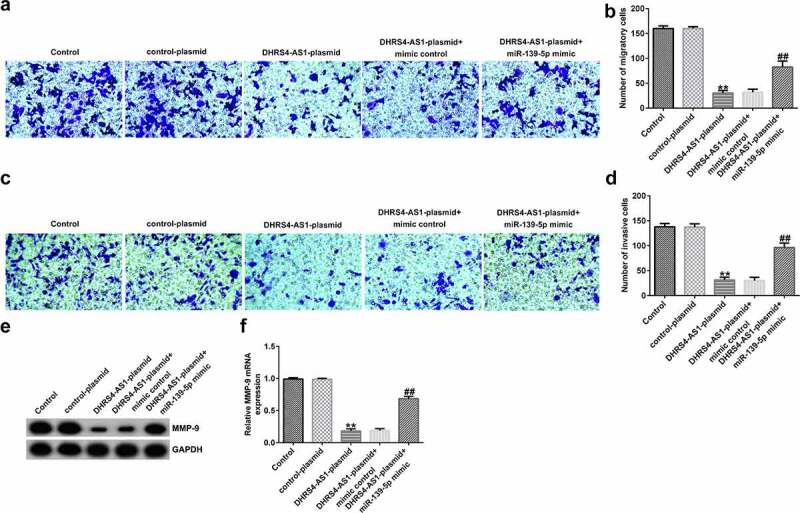
DHRS4-AS1 suppresses the migration and invasion of EC-ESCs by inhibiting miR-139-5p expression. (a) Representative images of the migration of EC-ESCs following transfection with DHRS4-AS1 and miR-139-5p, as determined by Transwell assay (bar = 100 µm). (b) The calculated number of migratory cells has been presented. (c) Transwell assay was performed to elucidate EC-ESC invasion in different groups, and representative images have been presented (bar = 100 µm). (d) The calculated number of invasive cells has been presented. MMP-9 (e) protein and (f) mRNA expression levels were determined via western blot analysis and reverse transcription-quantitative polymerase chain reaction, respectively. **P < 0.01 vs. control plasmid. ##P < 0.01 vs. DHRS4-AS1 plasmid + mimic control. DHRS4-AS1, DHRS4 antisense RNA 1; EC-ESCs, ectopic endometrial stromal cells; miR, microRNA.

As presented in Figure 5a–d, the migration and invasion of EC-ESCs was suppressed following DHRS4-AS1 plasmid transfection, compared to the control plasmid group; however, the observed decrease in migration and invasion triggered by DHRS4-AS1 plasmid transfection was reversed following co-transfection with the miR-139-5p mimic. The levels of MMP-9, a migration- and invasion-related protein, were determined. As shown in Figure 5eandf, the mRNA and protein expression levels of MMP-9 reduced remarkably in the DHRS4-AS1 plasmid group, compared to the control plasmid group. However, co-transfection with the miR-139-5p mimic increased MMP-9 expression level, compared with that in the DHRS4-AS1 plasmid group.

### miR-139-5p promotes proliferation and inhibits apoptosis of EC-ESCs

**Figure 6.**
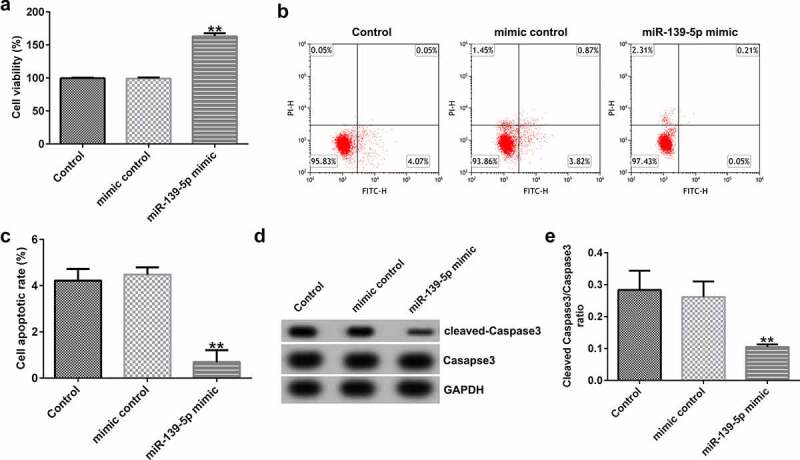
miR-139-5p promotes the proliferation and inhibits the apoptosis of EC-ESCs. (a) The viability of EC-ESCs was determined via MTT assay. (b and c) Apoptosis rate of EC-ESCs was evaluated via flow cytometry. (d) Expression levels of apoptosis-related proteins, including cleaved caspase-3 and caspase-3, were quantified in different groups. (e) Expression ratio of cleaved caspase-3/caspase-3 was calculated. **P < 0.01 vs. mimic control. EC-ESCs, ectopic endometrial stromal cells; miR, microRNA.

To investigate the effects of miR-139-5p in EC-ESCs proliferation and apoptosis, EC-ESCs were transfected with mimic control or miR-139-5p mimic. As shown in Figure 6, compared with the mimic control group, miR-139-5p mimic significantly enhanced EC-ESC viability (Figure 6a), reduced cell apoptosis (Figure 6bandc), and inhibited cleaved-Caspase 3 protein expression and the ratio of cleaved-Caspase 3/Caspase 3 (Figure 6dande).

**Figure 7.**
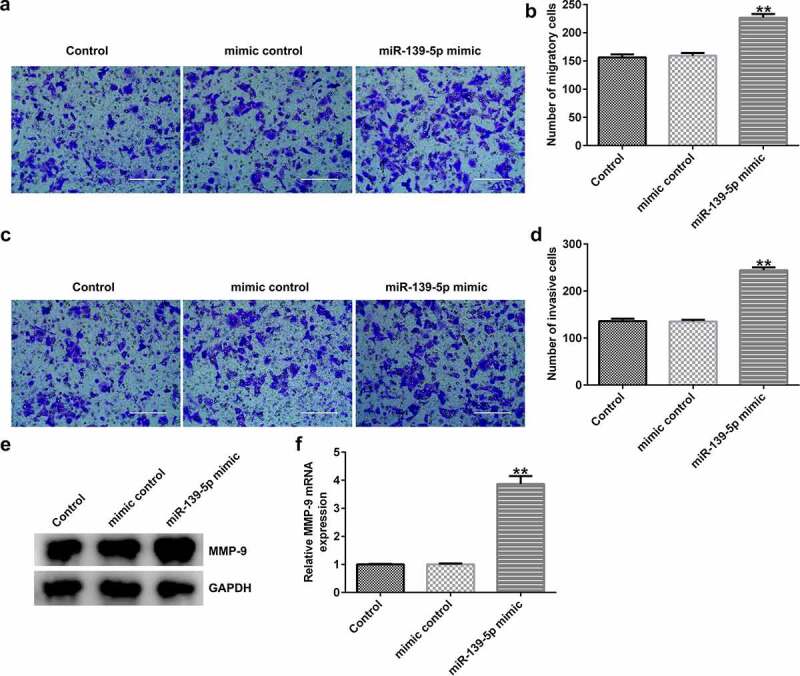
miR-139-5p enhances the migration and invasion of EC-ESCs. (a) Representative images of the migration of EC-ESCs following transfection with miR-139-5p mimic, as determined by Transwell assay (bar = 200 µm). (b) The calculated number of migratory cells has been presented. (c) Transwell assay was performed to elucidate EC-ESC invasion in different groups, and representative images have been presented (bar = 200 µm). (d) The calculated number of invasive cells has been presented. MMP-9 (e) protein and (f) mRNA expression levels were determined via western blot analysis and reverse transcription-quantitative polymerase chain reaction, respectively. **P < 0.01 vs. mimic control. EC-ESCs, ectopic endometrial stromal cells; miR, microRNA.

*miR-139-5p enhances EC-ESC migration and invasion*. To investigate the effects of miR-139-5p in EC-ESC migration and invasion, Transwell assay was performed. The data indicated that compared with the mimic control group, miR-139-5p mimic significantly enhanced EC-ESC migration (Figure 7aandb) and invasion (Figure 7candd), and promoted MMP-9 protein and mRNA expression (Figure 7eandf) in EC-ESCs.

### ARRDC3 is a target gene of miR-139-5p

**Figure 8.**
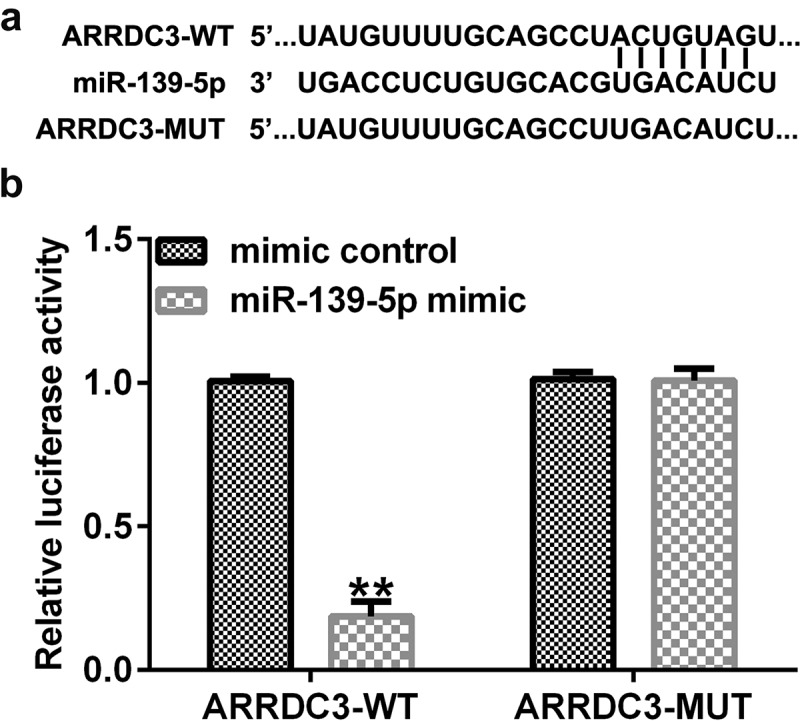
ARRDC3 is a downstream target of miR-139-5p. (a) The binding sites between miR-139-5p and ARRDC3 were predicted using TargetScan software. (b) A dual-luciferase reporter assay was performed to confirm the targeted relationship between miR-139-5p and ARRDC3. **P < 0.01 vs. mimic control. ARRDC3, arrestin domain containing 3; miR, microRNA.

Using the TargetScan online bioinformatics tool, putative binding sites between ARRDC3 and miR-139-5p were identified (Figure 8a). A dual-luciferase reporter assay was performed to validate the relationship between miR-139-5p and ARRDC3. As presented in Figure 8b, luciferase activity was significantly decreased in the miR-139-5p mimic and ARRDC3-wt co-transfection group compared with that in the mimic control and ARRDC3-wt groups. Furthermore, no significant differences were observed between the ARRDC3-mut groups. Collectively, these results confirm the relationship between miR-139-5p and ARRDC3.

## Discussion

Endometriosis, the cause of dysmenorrhea, chronic pelvic pain, and infertility, is defined as the presence and active growth of endometrial tissue outside the uterine cavity [36]. Endometriotic tissues and cells have biological functions similar to those of malignant cells, such as abnormal proliferation, invasion, metastasis, and apoptosis resistance [37]. The main differences between EC-ESCs and normal ESCs are enhanced migration and invasion, overgrowth, and enhanced epithelial-mesenchymal transition [38]. Endometriosis has a high recurrence rate, even with the complete surgical removal of lesions [39]. Additionally, the mechanism of recurrence remains unknown. It has been generally considered that the development and recurrence of endometriosis are associated with the proliferation, infiltration, metastasis, and other tumor-like biological behaviors of lesions [39]. No early diagnostic tools or radical treatments have been developed for this disease. The reason for this phenomenon is that the symptoms and complexity of its pathogenesis are diverse. Therefore, identifying novel therapeutic targets for endometriosis is crucial.

Additionally, lncRNAs and miRNAs are involved in diverse biological processes such as cell proliferation, differentiation, chromosome remodeling, epigenetic regulation, transcriptional modification, and post-transcriptional modification [40]. Moreover, miR-139-5p has been determined to be a pivotal regulator in diverse diseases [41–49]. A previous study reported that miR-139-5p is abundantly expressed in endometriosis [22]. Consistent with this study, our results showed that miR-139-5p expression was upregulated in endometriotic tissues and cells. Moreover, the upstream and downstream targets of miR-139-5p were predicted using StarBase and TargetScan and confirmed using a dual-luciferase reporter assay. The results revealed that DHRS4-AS1 and ARRDC3 have a targeted relationship with miR-139-5p.

Accumulating evidence suggests that lncRNAs may act as miRNA sponges at the post-transcriptional level, repressing miRNA expression and regulating mRNA expression at the post-transcriptional level [50,51]. In the present study, after confirming that DHRS4-AS1 is an upstream target of miR-139-5p, a significant downregulation of DHRS4-AS1 expression was detected in endometriotic tissues and cells. *In vitro* experiments further demonstrated that DHRS4-AS1 inhibited EC-ESC proliferation, migration, and invasion and promoted apoptosis by modulating miR-139-5p. The target gene of miR-139-5p was predicted to understand the molecular mechanism by which miR-139-5p regulates the behavior of EC-ESCs. We ascertained that ARRDC3 is a direct target of miR-139-5p. As ARRDC3 plays a vital role in regulating cell proliferation, migration, and invasion [52,53], miR-139-5p may regulate EC-ESC proliferation, migration, invasion, and apoptosis by regulating ARRDC3 expression. To our knowledge, the current study is the first to comprehensively examine the expression and function of DHRS4-AS1 in endometriosis. In addition, the present study is the first to reveal a relationship between lncRNA DHRS4-AS1 and miR-139-5p. Importantly, the current study obtained EC-ESCs using a previously described method to serve as an *in vitro* cell model for endometriosis, which conferred additional reliability to the results of the current study.

It is worth noting that the current study has several limitations. First, the clinical sample size for endometriosis was small; therefore, the reliability of the results would have been improved by expanding the sample size. Additionally, a control group of patients with uterine fibroids was utilized instead of healthy women. An *in vivo* model was not constructed for these experiments. In future studies, these limitations will be addressed by further expanding the number of samples and refining the experimental protocols to further understand the mechanism of the DHRS4-AS1/miR-139-5p/ARRDC3 axis in endometriosis. The correlation between lncRNA DHRS4-AS1 expression and clinicopathological parameters of patients with endometriosis will be investigated in a future study.

In summary, this study is the first to reveal that DHRS4-AS1 is involved in endometriosis through regulating EC-ESC proliferation, migration, invasion, and apoptosis via the miR-139-5p/ARRDC3 axis (Supplementary Figure 1).

## Conclusion

These results demonstrated that lncRNA DHRS4-AS1 may protect against the development of endometriosis by regulating the miR-139-5p/ARRDC3 axis, providing a novel target for the treatment of endometriosis.

## Supplementary Material

Supplemental Material

## Research highlights

1. DHRS4-AS1 expression levels decreased, while miR-139-5p expression levels increased in endometrial tissues and cells.
2. DHRS4-AS1 inhibits proliferation and promotes apoptosis of EC-ESCs by targeting miR-139-5p.
3. DHRS4-AS1 suppresses EC-ESC migration and invasion by sponging miR-139-5p.
4. ARRDC3 is a target gene of miR-139-5p.

## Statement of Availability of Data

The datasets used and/or analyzed during the present study are available from the corresponding author on reasonable request.
