# Supplementary figures and images for: Long non-coding RNA DHRS4 antisense RNA 1 inhibits ectopic endometrial cell proliferation, migration, and invasion in endometriosis by regulating microRNA-139-5p expression
Source: Bioengineered. 2022 Apr 12;13(4):9792–804. doi: 10.1080/21655979.2022.2060781 (PMC9161999; doi:10.1080/21655979.2022.2060781)

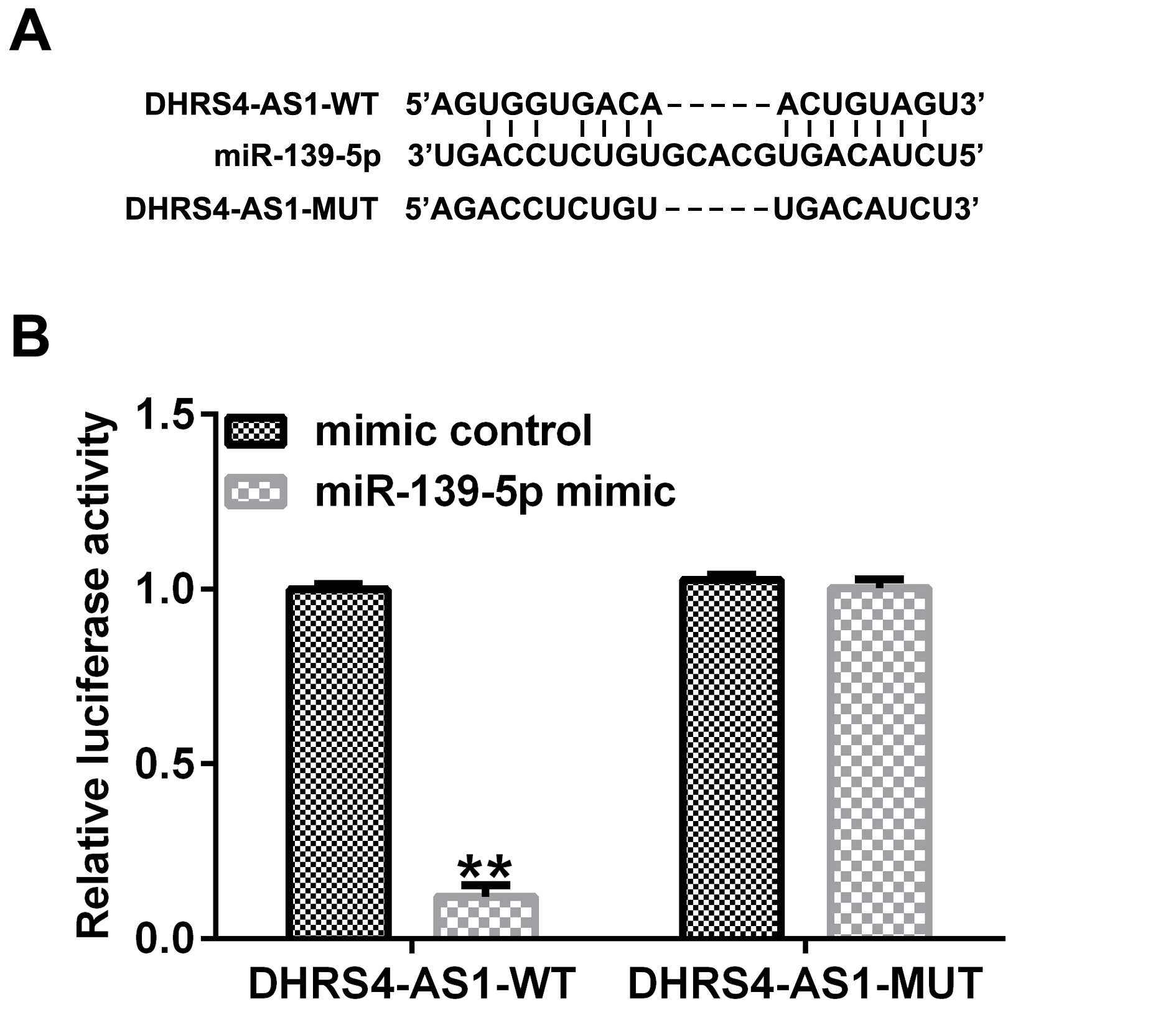

Supplement: Supplemental Material [file KBIE_A_2060781_SM6609.tif]
